# Left ventricular mechanical dysfunction in diet-induced obese mice is exacerbated during inotropic stress: a cine DENSE cardiovascular magnetic resonance study

**DOI:** 10.1186/s12968-015-0180-7

**Published:** 2015-08-27

**Authors:** Christopher M. Haggerty, Andrea C. Mattingly, Sage P. Kramer, Cassi M. Binkley, Linyuan Jing, Jonathan D. Suever, David K. Powell, Richard J. Charnigo, Frederick H. Epstein, Brandon K. Fornwalt

**Affiliations:** Saha Cardiovascular Research Center, University of Kentucky, Lexington, KY USA; Department of Pediatrics, University of Kentucky, Lexington, KY USA; College of Medicine, University of Kentucky, Lexington, KY USA; Department of Physiology, University of Kentucky, Lexington, KY USA; Department of Biomedical Engineering, University of Kentucky, Lexington, KY USA; Department of Biostatistics, University of Kentucky, Lexington, KY USA; Departments of Biomedical Engineering and Radiology, University of Virginia, Charlottesville, VA USA; Geisinger Health System, Institute for Advanced Application, 100 North Academy Avenue, Danville, PA 17822 USA

**Keywords:** Cardiovascular magnetic resonance, DENSE, Strain, Stress, Mice, Obesity

## Abstract

**Background:**

Obesity is a risk factor for cardiovascular disease. There is evidence of impaired left ventricular (LV) function associated with obesity, which may relate to cardiovascular mortality, but some studies have reported no dysfunction. Ventricular function data are generally acquired under resting conditions, which could mask subtle differences and potentially contribute to these contradictory findings. Furthermore, abnormal ventricular mechanics (strains, strain rates, and torsion) may manifest prior to global changes in cardiac function (*i.e.*, ejection fraction) and may therefore represent more sensitive markers of cardiovascular disease. This study evaluated LV mechanics under both resting and stress conditions with the hypothesis that the LV mechanical dysfunction associated with obesity is exacerbated with stress and manifested at earlier stages of disease compared to baseline.

**Methods:**

C57BL/6J mice were randomized to a high-fat or control diet (60 %, 10 % kcal from fat, respectively) for varying time intervals (*n* = 7 – 10 subjects per group per time point, 100 total; 4 – 55 weeks on diet). LV mechanics were quantified under baseline (resting) and/or stress conditions (40 μg/kg/min continuous infusion of dobutamine) using cine displacement encoding with stimulated echoes (DENSE) with 7.4 ms temporal resolution on a 7 T Bruker ClinScan. Peak strain, systolic strain rates, and torsion were quantified. A linear mixed model was used with Benjamini-Hochberg adjustments for multiple comparisons.

**Results:**

Reductions in LV peak longitudinal strain at baseline were first observed in the obese group after 42 weeks, with no differences in systolic strain rates or torsion. Conversely, reductions in longitudinal strain and circumferential and radial strain rates were seen under inotropic stress conditions after only 22 weeks on diet. Furthermore, stress cardiovascular magnetic resonance (CMR) evaluation revealed supranormal values of LV radial strain and torsion in the obese group early on diet, followed by later deficits.

**Conclusions:**

Differences in left ventricular mechanics in obese mice are exacerbated under stress conditions. Stress CMR demonstrated a broader array of mechanical dysfunction and revealed these differences at earlier time points. Thus, it may be important to evaluate cardiac function in the setting of obesity under stress conditions to fully elucidate the presence of ventricular dysfunction.

**Electronic supplementary material:**

The online version of this article (doi:10.1186/s12968-015-0180-7) contains supplementary material, which is available to authorized users.

## Background

Obesity is a highly prevalent disease [1, 2] that is strongly associated with increased mortality, primarily due to cardiovascular disease [3]. The linkage between obesity and cardiovascular-related mortality is multi-factorial: obesity carries risk factors for atherosclerotic disease, but there are risk factors independent of atherosclerosis as well [4], potentially including direct effects on the heart [5, 6]. As a possible consequence of these direct effects, there is mounting evidence of decreased left ventricular (LV) cardiac function in the setting of obesity for both humans [7–9] and mouse models [10–12]. However, numerous studies have observed no dysfunction in obesity [13–18], so this issue remains controversial.

One potential explanation for these inconsistent reports is the fact that most assessments of cardiac function are performed under baseline resting conditions, when the heart is under comparatively little metabolic demand. Under these conditions, subtle functional differences that manifest with physical exertion or stress may be masked and difficult to detect. For example, Calligaris *et al.* found that fractional shortening and maximum time rates of pressure development (dP/dt) were no different between control and obese mice under baseline conditions, but the contractile response in the obese subjects was reduced with pharmacologic stress [11].

Another potential cause for the contradictory findings surrounding cardiac function in the setting of obesity is the difference in functional end points studied. Specifically, the majority of studies that rely on measures like ejection fraction or fractional shortening to characterize cardiac function [13–18] report no dysfunction associated with obesity, while the majority of studies that quantify cardiac strains (or other similar measures of ‘cardiac mechanics’) [7–10, 19] report the presence of systolic dysfunction with obesity. Of the two approaches, strain-based measures are widely regarded as being more sensitive measures of function, as evidenced by the findings of altered strains in patients with heart failure with preserved ejection fraction (HFpEF) [20, 21]. Furthermore, LV strains have been shown to be superior to ejection fraction for predicting outcomes in patients with cardiovascular disease [22, 23].

Using sensitive measures of cardiac mechanics, the objectives of the present study were therefore: 1) to characterize the evolution of systolic cardiac function (or dysfunction) in mice in response to chronic high-fat feeding and in relation to concurrent assessments of systemic blood pressure, glucose tolerance, and cardiac remodeling; and 2) to compare cardiac function measures between baseline and inotropic stress conditions over time. We hypothesized that cardiac mechanics would progressively worsen in obese mice in association with the development of other obesity co-morbidities, and that inotropic stress would exacerbate this cardiac dysfunction and reveal dysfunction at earlier stages of disease.

## Methods

### Animals and diets

All animal procedures conformed to the United States Public Health Service policies for the humane care and use of animals, and all procedures were approved by the institutional animal care and use committee at the University of Kentucky. Male C57Bl/6 mice were purchased from the Jackson Laboratory (Bar Harbor, ME) and fed either a high-fat diet of 60 % calories from fat *ad libitum* (Research Diets #D12492), or a low fat control diet with 10 % calories from fat, *ad libitum* (Research Diets #D12450B or #D12450J). Animals were group housed in ventilated cages in a temperature-controlled room with a 14:10 light:dark cycle and provided with nesting material (Nestlets® and Enviro-dry®).

### Experiment 1 – longitudinal study of the effects of chronic high-fat feeding and obesity co-morbidities on cardiac mechanics

A single set of 20 mice (“Cohort #1”; *n* = 10 per diet group) were longitudinally studied with measures of blood pressure, glucose tolerance, cardiac function and mechanics using cardiovascular magnetic resonance (CMR), and body composition. For this experiment, CMR only interrogated baseline cardiac function, with no beta-adrenergic agonism. Tests were performed on alternating intervals through 54 weeks on diet, as detailed in Table 1. A separate set of 20 mice (“Cohort #2”; *n* = 10 per diet group) was also studied using glucose tolerance and baseline CMR measurements after 3 and 4 weeks on diet, respectively. This second set was added in order to provide 1) a measure of glucose tolerance at an earlier time point (3 weeks on diet) and 2) supplemental data at the 4 week CMR measurement to negate a group-wise difference in heart rate (which could have affected loading conditions and therefore peak strains) in the first set of mice.

**Table 1:** Details of study cohorts and measurements

	Measurement	Time Point (weeks on diet)
Cohort #1	Blood Pressure	2^a^, 5, 9, 13, 17, 21, 25, 29, 33, 37^b^, 41^b^, 53^b^
	Glucose Tolerance	7, 11, 15, 19, 23, 27, 32, 36^b^, 40^b^, 52^b^
	Body Composition	19, 23, 27, 32, 36^b^, 40^b^, 52^b^
	CMR at baseline	4, 10, 16, 22, 28, 34^b^, 42^b^, 54^b^
	CMR with stress	55^b^
Cohort #2	Glucose Tolerance	3
	CMR at baseline	4
	CMR with stress	22, 28
Cohort #3	CMR with stress	4
Cohort #4	CMR with stress	10
Cohort #5	CMR with stress	16

All cohorts comprised on 20 subjects (*n* = 10 per group); ^a^Procedure Acclimatization, Data not reported; ^b^ Reduced numbers in obese group from attrition

### Experiment 2 – effects of inotropic stress on cardiac mechanics in obesity

CMR studies were performed at 4, 10, 16, 22, 28, and 55 weeks on diet under maximal inotropic stress conditions (intraperitoneal infusion of 40 μg/kg/min dobutamine) [24]. Independent groups of 20 mice (*n* = 10 per diet) were used for each time point, with the exception of 1 group (“Cohort #2”; See Table 1), which was scanned at both 22 and 28 weeks on diet. Note that the Cohort #1 mice were also used for the 55-week measurement (with *n* = 7 in the obese group with attrition). In total, 100 animal subjects were used for both experiments.

### Blood pressure

Conscious systolic blood pressure was measured using a volume and pressure-recording tail cuff (CODA System, Kent Scientific, Torrington, CT) [25]. To minimize stress, the animals were pre-conditioned to measurement-associated handling prior to study data collection (Week 2), and measurements were made in a quiet room with only the usual caretaker present. Data were acquired over five consecutive days at the same time of day during the light cycle. For a given day, at least 5 passing measurements (of 20) were required to retain the average systolic pressure for that day. Criteria for acceptance were: 1) the ‘pass/fail’ analysis built into the CODA system software; 2) a value less than 200 mmHg, unless it was within 1 standard deviation of the remaining passing values; and 3) a value greater than 50 mmHg, unless it was within 1 standard deviation of the remaining passing values. Finally, if the standard deviation of the remaining passing values was greater than 30 mmHg, the data for that day were discarded [25]. Successful readings on at least three of five days were required to report a value for a given week, which represented the average of the individual day averages.

### Glucose tolerance

Mice were fasted for 6 h in clean cages and injected intraperitoneally with glucose (1 g/kg body weight). Blood glucose concentrations were measured with a Breeze2 meter (Bayer HealthCare AG, Leverkusen, Germany) at 0 (this measurement defined as the fasting blood glucose), 15, 30, 60, and 120 min after injection. The area under the glucose concentration curve (AUC) was calculated after subtracting the area below the fasting baseline.

### Body composition

Whole body composition (fat mass, lean mass, and water) was measured with a commercial system (model 100; EchoMRI LLC, Houston, TX). These quantities were used to derive the percentage of fat mass in total body mass, as well as the ratio of lean-to-fat mass percentages.

### CMR

**Fig. 1:**
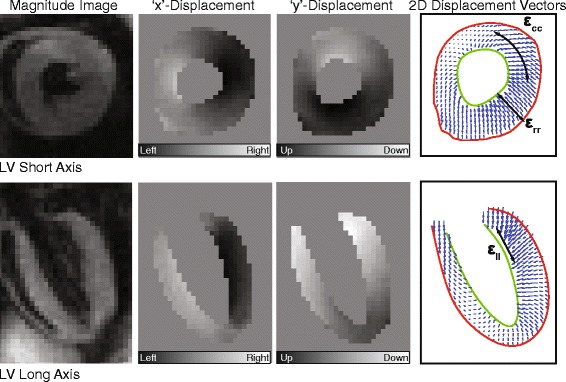
Representative DENSE CMR images of a mouse left ventricle (LV) in the short (*top*) and long axis (*bottom*) orientations at peak systole. The far right panel demonstrates the corresponding vector displacement field that can be derived from the phase displacement images (middle panels), along with the corresponding decomposition into radial (ε_rr_), circumferential (ε_cc_), and longitudinal (ε_ll_) strains

CMR was performed on a 7-Tesla Bruker ClinScan system (Bruker, Ettlingen, Germany) equipped with a 4-element phased array cardiac coil and a gradient system with a maximum strength of 450 mT · m^−1^ and a maximum slew rate of 4500 mT · m^−1^ · s^−1^. CMR images were acquired using the tissue motion-sensitive method known as cine Displacement Encoding with Stimulated Echoes (DENSE) [26–28]. In this sequence, a displacement encoding pulse is applied immediately after the detection of an electrocardiogram R-wave (and during the exhalation phase of the respiratory cycle), which marks the depolarization of the ventricles and the onset of contraction. To null signal from fat, a fat-saturating pulse is applied following the previous R-wave trigger, before the encoding pulse. The encoding pulse, which consists of both radiofrequency and gradient pulses, stores the position-encoded longitudinal magnetization. Through subsequent, successive applications of a readout module (consisting of a radiofrequency excitation pulse, a displacement decoding gradient, and an interleaved spiral k-space trajectory) the phase component of the MR signal directly encodes the pixel-wise tissue displacement as a function of the cardiac cycle. In this way, a total of 12–20 frames per cardiac cycle (dependent on heart rate; repetition time = 7.4 ms) were acquired with orthogonal in-plane (*i.e.*, ‘x’ and ‘y’) displacement encoded images and the magnitude image. Examples of these images for a representative mid-ventricular short-axis slice and long-axis 4-chamber slice are shown in Fig. 1. Other relevant acquisition parameters included: field of view = 32 mm, matrix = 128 × 128, slice thickness = 1 mm, echo time = 1 ms, number of averages = 2, number of spiral interleaves = 36 (1 leave acquired per heartbeat), and displacement encoding frequency = 0.8–1.0 cycles/mm.

For the baseline CMR studies, 3 LV short-axis and 2 LV long-axis DENSE images were acquired. The long-axis images consisted of a standard apical 4-chamber view and a 2-chamber view, perpendicular to the 4-chamber. The short-axis images were planned perpendicular to the 4-chamber image such that the mid-ventricular slice was shifted 50 % of the endocardial, end-systolic long-axis length from the apex; the basal and apical slices were positioned 20 % of that total length above and below the mid-ventricular slice, respectively (range: 1–1.4 mm).

For stress CMR scans, dobutamine was infused into the peritoneal cavity using an MRI compatible syringe pump (Harvard Apparatus, Holliston, MA) for 10–20 min (depending on heart rate response) following slice planning and baseline acquisitions, but prior to and continuously during acquisition of stress DENSE. Three LV short-axis and 4-chamber DENSE images were acquired with the same slice planning as described for baseline studies.

### Physiologic monitoring during CMR scans

Anesthesia was induced with isoflurane using a precision vaporizer delivering 1.5–2.5 % isoflurane in oxygen at a rate of 1 L · min^−1^. Three legs were shaved for placement of cutaneous electrocardiogram electrodes required for cardiac-gated imaging. During the scan, anesthesia was maintained with 6–10 % desflurane in oxygen at a flow rate of 0.2–0.5 L · min^−1^. A diaphragm was placed on the abdomen to monitor breathing and to gate image acquisition to respiration (in addition to the cardiac cycle). A rectal thermometer was used to monitor core temperature, which was maintained between 36.3 °C and 37.3 °C during the procedure using a heated water system. All vital signs were continuously monitored with a fiber optic system (SA Instruments, Inc., Stony Brook, NY).

### Image processing

The DENSE phase images were used to derive quantitative measures of cardiac mechanics using custom DENSE analysis software in MATLAB (The Mathworks, Inc., Natick, MA). The basic steps included semi-automatic motion-guided segmentation of the myocardium, phase unwrapping, spatial smoothing, temporal fitting of displacements, and calculations of strain, strain rate, torsion, and synchrony [29–31].

Strains were quantified with the 2-dimensional Lagrangian finite strain tensor. For radial and circumferential strains (defined with respect to the LV center of mass), the three short-axis images were partitioned based on the standardized American Heart Association 16 segment model and a strain vs. time curve was calculated for each segment. A mean curve was defined by averaging these segment curves; the peak of the mean curve represents the peak strain reported. Peak longitudinal strain was similarly defined from the long-axis images and a defined coordinate system; however, the apical segments covering the bottom one-third of the ventricle were excluded from the analysis. Peak strain rates were calculated by taking positive and negative peaks of the first temporal derivative of the mean strain curve. All strains are reported as positive magnitudes to facilitate visual comparisons. Peak torsion [° · cm^−1^] was defined as the maximal slope of a linear regression of instantaneous, spatially averaged myocardial twist angles as a function of the distance between the three short-axis slices.

To quantify left ventricular volumes, mass, and ejection fraction, the DENSE magnitude images were segmented at end-diastole (taken as the last frame of the cardiac cycle because of insufficient nulling of the blood pool signal in the first image frame) and end-systole. These contours were inputted to a custom 3-dimensional surface fitting algorithm [32] to calculate the volume and mass data. Briefly, thin-plate spline interpolation was used to fit a smooth surface to the defined boundary and valve points. Myocardial density was assumed to be 1.05 g/mL.

### Statistics

Blood pressure, glucose tolerance, and body composition results were interpolated to be defined at the same time points as the CMR-derived outcome variables. Linear mixed models were then fit to relate outcome variables to time and group, allowing each group to have its own temporal trend and not assuming a specific parametric form (*e.g.*, linear) for that trend. For the longitudinal measurements in experiment 1, random effects were included to account for correlations among repeated measurements on the same specimen. The groups’ temporal trends were tested for equality; if this null hypothesis was rejected, then post-hoc tests were performed to compare the groups at various time points using a Benjamini-Hochberg adjustment for multiple comparisons. Some models were also fit with explanatory variables other than group (*e.g.*, LV mass, glucose AUC, and body fat%). Version 9.3 of SAS software (SAS Institute, Cary, NC) was employed for these data analyses. T-tests were used, as indicated, for select individual comparisons. Pearson correlation coefficients were computed to report associations between peak strain and other obesity co-morbidities. The significance level was set to 0.05.

Continuous variables are reported in the text as mean ± standard deviation. Where applicable, data are graphically represented using box-and-whisker plots in which the median is represented by a single horizontal line, the box represents the inter-quartile range, and the whiskers represent the range of the data.

## Results

### Experiment 1- longitudinal study of development of obesity and co-morbidities (*n* = 20)

**Fig. 2:**
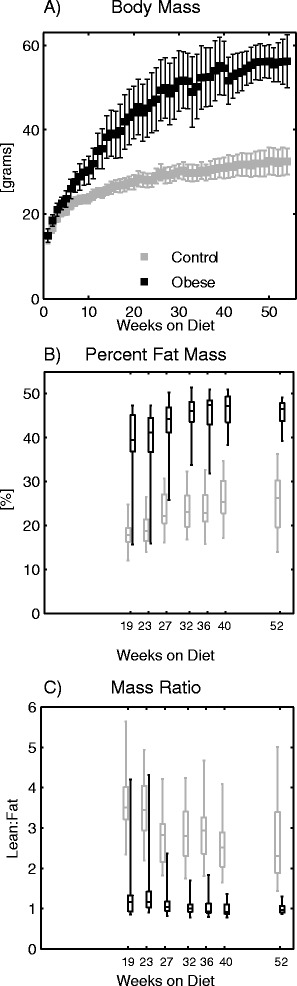
**a** Total body mass, (**b**) percent of fat mass, and (**c**) ratio of lean mass to fat mass percentages for the obese and control mice at the indicated times with respect to diet initiation. The obese group had significantly higher body mass (from week 2, onward) and percentage of fat mass with a lower lean:fat ratio (from earliest measurement at week 19)

Mice fed the high-fat diet had significantly higher body mass by the time of the first CMR scan after 4 weeks on the diet (*p* = 0.0066 at that time point, *p* < 0.0001 overall; Fig. 2a), and this separation increased substantially with time. By study conclusion (54 weeks on diet), the high-fat (obese) group weighed 56.1 ± 5.7 g as compared to 32.5 ± 3.5 g for the low-fat controls. The obese mice also had significantly higher percentage of fat mass (Fig. 2b; 45.5 ± 3.4 % vs. 25.2 ± 6.7 % at week 52) and a significantly lower lean:fat mass percentage ratio (Fig. 2c; 1.01 ± 0.15 vs. 2.69 ± 1.07 at week 52) compared to the control mice.

**Fig. 3:**
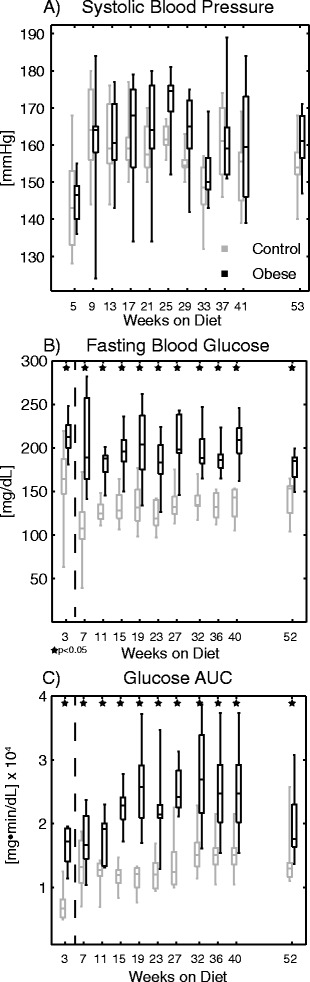
Data for (**a**) systolic blood pressure, (**b**) fasting blood glucose, and (**c**) glucose clearance area under the curve (AUC) at the indicated times with respect to diet initiation. Differences in blood pressure were not statistically significant, while both measures of glucose tolerance were significantly different throughout the study (overall and at each individual time point)

Conscious systolic blood pressure measurements via tail cuff yielded a passing result for each mouse at each measurement week. The data (Fig. 3a) displayed a diverging trend in weeks 17–29 with the obese group having higher pressure, but the overall linear mixed model did not report significant differences (*p* = 0.13).

Measures of glucose regulation, both with respect to fasting glucose levels (Fig. 3b) and the glucose tolerance test area under the curve (AUC; Fig. 3c), were significantly different (worse in obese group; *p* < 0.0001 overall for both measures) throughout the study from the earliest evaluation. Separate experiments at week 3 (left of dashed line in Fig. 3b and c) demonstrated that glucose intolerance developed prior to the first CMR time point (*p* < 0.05 via *t*-test).

Finally, three subjects from the obese group died during the study (during weeks 34, 41, and 43 respectively), compared to zero in the control group. The causes of mortality could not be determined.

### Obesity is associated with impaired LV peak strains but preserved ejection fraction at baseline

**Fig. 4:**
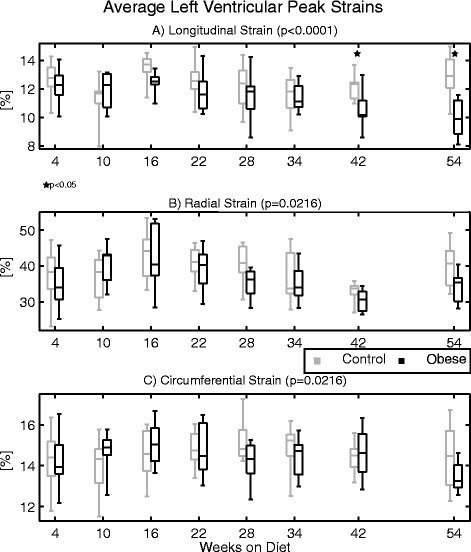
Average left ventricular peak strains at baseline in the (**a**) longitudinal, (**b**) radial, and (**c**) circumferential directions at the indicated times with respect to diet initiation from experiment 1. All strains are reported as positive to facilitate visual comparison (*i.e.* lower strains imply impaired function). Linear mixed models reported significant differences between groups for all strain measures over time; however, only longitudinal strain was reduced in the obese group at individual time points by post-hoc analyses (denoted by stars)

Figure 4 compares the average LV peak strains at baseline between groups at each measurement (see also Additional file 1: Table S1). With the exception of the primary experimental set of mice at the 4-week measurement (necessitating the inclusion of the second set, as discussed in the methods), there were no significant differences in cardiac period between groups at any time point (see Additional file 1: Table S2). The linear mixed models analysis reported a statistically significant time*group interaction for all strain dimensions (p-values reported in the figure). However, after correcting for multiple comparisons, post-hoc differences between groups at individual time points were only observed for peak longitudinal strain, for which strains in the obese group were significantly less than controls at weeks 42 and 54.

There were no statistically significant differences in peak strain rates (systolic or diastolic) or peak torsion between groups over time (Additional file 1: Table S3-S5).

**Fig. 5:**
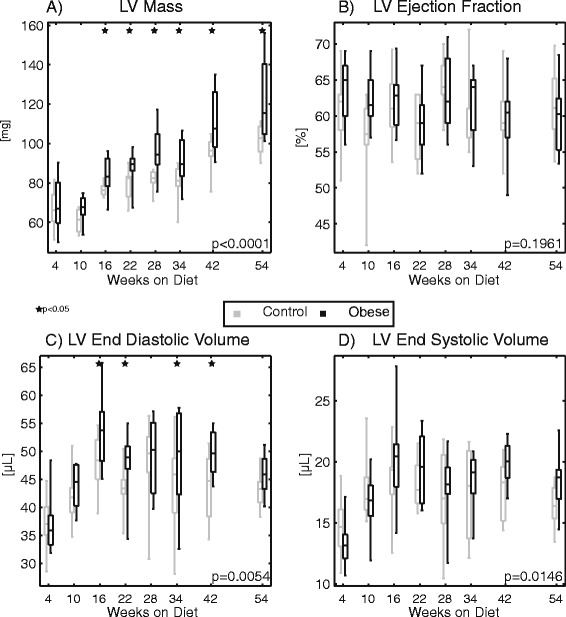
Plots of (**a**) left ventricular mass, (**b**) ejection fraction, (**c**) end diastolic volume, and (**d**) end systolic volume derived from the DENSE magnitude images at the indicated times with respect to diet initiation. Mass and volumes were significantly elevated overall in the obese group (individual time point significant differences indicated by stars), but there was no significant difference in ejection fraction

Ventricular mass and volume data are shown in Fig. 5. LV mass (Fig. 5a) progressively increased throughout the study for both groups, but was significantly higher in the obese group. This difference was first detected after 16 weeks on diet and persisted through the remainder of the study. End-diastolic and end-systolic volumes (Fig. 5c and d) were similarly higher for the obese group, although significant individual time point differences were only observed for end-diastolic volumes. Despite these differences in mass and volumes, there was *no significant difference in ejection fraction* between groups (*p* = 0.196; Fig. 5b).

### Association of LV peak strains with obesity co-morbidities

Table 2 presents Pearson correlation coefficients (unadjusted) and linear mixed model p-values for LV mass, glucose AUC, and body fat % in relation to LV peak strains, adjusted for group. Both longitudinal and radial strains had weak-to-moderate correlation coefficients with each secondary metric; however, after adjusting for group, only associations between myocardial mass and radial strain, as well as glucose AUC and radial strain were statistically significant. Body fat % was not significantly associated with any strain measure after adjusting for group.

**Table 2:** Associations^a^ of obesity sequelae with LV peak strains

	Myocardial mass	Glucose AUC	Fat% of body mass
Longitudinal Strain	*R* = −0.34;	*R* = −0.24;	*R* = −0.47;
	*p* = 0.407	*p* = 0.065	*p* = 0.207
Radial Strain	*R =* −0.32;	*R* = −0.22;	*R* = −0.45;
	*p* = *0.025*	*p* = *0.037*	*p* = 0.240
Circumferential Strain	*R* = −0.03	*R* = −0.05;	*R* = −0.25;
	*p* = 0.345	*p* = 0.091	*p* = 0.305

^a^Values reported represent un-adjusted Pearson correlation coefficients (R), and p-values from linear mixed model adjusted for group

### Experiment 2 – cardiac mechanics under dobutamine stress (*n* = 20 per time point)

**Fig. 6:**
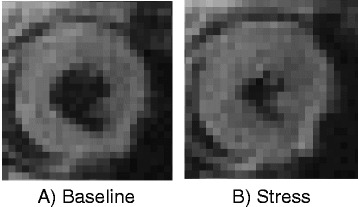
Representative mid-ventricular short-axis DENSE magnitude images at end-systole for (**a**) baseline and (**b**) stress imaging studies for a single subject. There is an apparent difference in chamber area between the two images, which is indicative of the change in contractility with stress

With dobutamine stress, heart rate increased from 441 ± 50 beats per minute to 571 ± 29 beats per minute. Increased contractility from baseline was evidenced by decreased end-systolic area (Fig. 6) and generally increased left ventricular strains (except longitudinal strain), strain rates, and torsion. There were no statistical differences in heart rate between groups at peak stress, with the exception of the 55-week measurement in which the obese group had a slightly blunted heart rate response compared to controls (577 ± 32 vs. 546 ± 25 beats per minute for controls vs. obese groups, respectively; *p* = 0.046 by *t*-test; Additional file 1: Table S2).

**Fig. 7:**
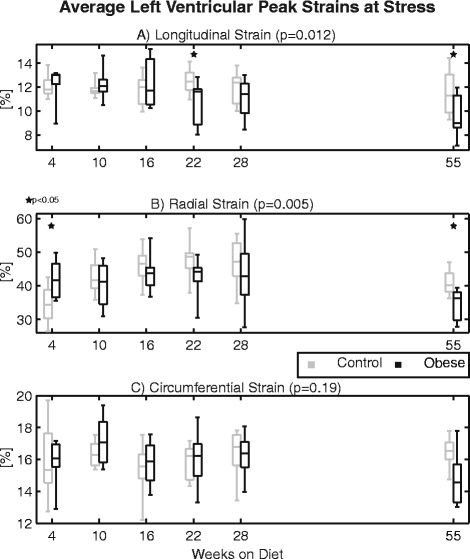
Average left ventricular peak strains at stress in the (**a**) longitudinal, (**b**) radial, and (**c**) circumferential directions at the indicated times with respect to diet initiation from experiment 2. All strains are reported as positive to facilitate visual comparison (*i.e.* lower strains imply impaired function). Statistically significant differences between groups by linear mixed models were observed with respect to longitudinal and radial strains over time, with post-hoc findings of individual time point differences denoted by stars. Longitudinal strain was reduced in the obese group at 22 and 55 weeks; radial strain was increased at week 4, but reduced by week 55

Figure 7 shows the cross-sectional comparison of peak strains under dobutamine stress for the control and obese groups with respect to time on diet. Based on linear mixed models analysis, there were overall statistically significant differences in longitudinal and radial, but not circumferential strains at peak stress over time. Post-hoc analysis demonstrated that longitudinal strains were significantly lower in the obese group at 22 and 55 weeks, whereas radial strains were increased in the obese group at week 4, but significantly lower by 55 weeks on diet (see Additional file 1: Table S6).

**Fig. 8:**
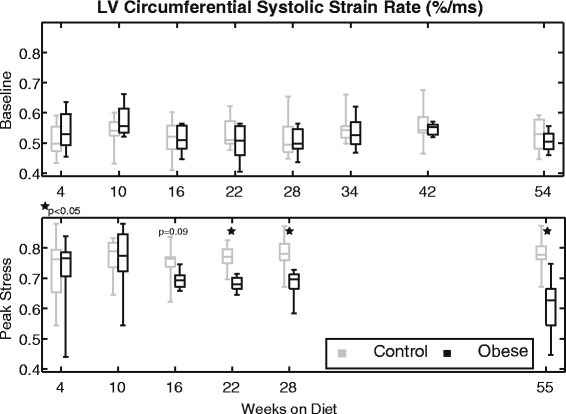
Left ventricular circumferential systolic strain rates at baseline (*top*) and stress (*bottom*) at the indicated times with respect to diet initiation. No statistical differences between groups were observed at baseline. Strain rates increased for both groups with stress; however, the obese group did not maintain this contractile reserve over time and, beginning at week 22, they had significantly lower values than controls

Figure 8 shows the peak circumferential systolic strain rates for the baseline (top) and stress (bottom) scans. While there were no statistical differences in circumferential systolic strain rates at baseline (*p* = 0.29), there was a statistically significant (*p* < 0.001) and progressive decline in circumferential strain rates for the obese mice at peak stress over time. Post-hoc analysis revealed that these differences were significant from 22 weeks onward, while there was a trend (*p* = 0.09) towards a separation as early as 16 weeks on diet. Similar trends were observed with respect to radial systolic strain rates at peak stress (overall, *p* < 0.001; see also Additional file 1: Table S7-S8)).

**Fig. 9:**
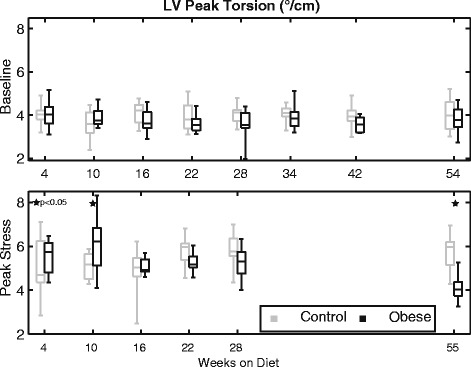
Left ventricular peak torsion at baseline (*top*) and stress (*bottom*) at the indicated times with respect to diet initiation. No statistical differences between groups were observed at baseline. Torsion at stress was generally increased compared to baseline, but this response was significantly different between groups over time by linear mixed models. Torsion was elevated in the obese group at week 10 but reduced in the obese group by week 55 via post-hoc analysis

Figure 9 shows peak torsion for the baseline (top) and stress (bottom) scans. There was no statistical difference between groups with respect to torsion at baseline (*p* = 0.30); however, there was a significant difference in torsion at stress between groups over time (*p* < 0.001; Additional file 1: Table S9). Similar to what was observed for radial strain, peak torsion was higher in the obese group early (week 10), but was ultimately decreased compared to controls by 55 weeks.

## Discussion

While the relationship between obesity and increased cardiovascular mortality is clear, the effects of obesity on cardiac function are less understood. Murine models have been used extensively to help address this shortcoming with conflicting results: many studies have reported decreased cardiac function [12, 33–36], while others have found no functional change [13, 14, 18, 37, 38], and some have even reported improved function [15]. This study sought to address and to help resolve these discrepancies by evaluating sensitive and reproducible CMR-based measures of cardiac mechanics at baseline and under inotropic stress.

The primary findings of this study are as follows. First, we found no significant difference in baseline ejection fraction between obese and control mice through a year of high-fat feeding, in agreement with many investigators reporting no functional change based on ejection fraction or fractional shortening. However, assessing cardiac mechanics (strain, strain rate, torsion) with DENSE CMR, we did see altered systolic function in the obese mice. At baseline, this dysfunction was characterized by decreased longitudinal strain after 42 weeks on diet; however, at peak stress, changes in strains, strain rates, and torsion were all apparent, with dysfunction present by several measures after 22 weeks on diet. So even though baseline ejection fraction was normal, systolic dysfunction was still present with obesity, which is consistent with what has been reported in humans [39]. Since cardiac strains are also better predictors of mortality than ejection fraction [23], clinical assessments of cardiac function in obesity should incorporate measures of cardiac mechanics, like peak strains. In particular, peak longitudinal strain appears to be a critical assessment in obesity as it was a consistent discriminator between obese and control mice both at baseline and under stress in this study. There are also numerous human studies that have reported differences in longitudinal strain with obesity [8, 40–42]. From our data, the preferential change in longitudinal strain may be partially related to a relative alteration of the myocardial fiber arrangement with the modest dilation observed in the end-diastolic volume. Additionally, lipid infiltration and mild chronic ischemia and inflammatory signaling preferentially impacting the subendocardial and subepicardial layers have also been proposed as potential mechanisms to explain longitudinal strain changes [40]. Additional work is needed to elucidate the exact mechanisms.

### Cardiac mechanics at baseline vs. stress

The use of dobutamine stress in this study produced several meaningful insights as compared to the knowledge gained solely from evaluations at baseline. First, cardiac dysfunction was exacerbated with stress. While temporal differences in peak strains were observed at baseline, stress scanning additionally uncovered differences/deficiencies in systolic strain rates and torsion. In both of those cases, these differences appear to be the result of a loss of contractile reserve function in the obese group over time, as they were no longer able to increase strain rate or torsion with stress to the elevated level of the controls. Inhibitory interactions of insulin with the cardiac β_2_-adrenergic receptors may play a primary role in this blunted response [43]. These changes may also be the result of myocardial perfusion defects with stress, which have also been reported in these mice around the same time [44]. Additionally, cardiac dysfunction was detectable much earlier in the disease process at stress (22 weeks on diet) than at baseline (42 weeks). Notably, this earlier timeline is more consistent with findings of cardiac dysfunction from previous studies [10, 36, 44]. Finally, stress scans revealed a pattern of potentially early supranormal cardiac mechanics in the obese group that was not detected at baseline. Specifically, elevated radial strain and torsion in the obese mice compared to controls (weeks 4 and 10, respectively) are intriguing findings that are suggestive of complex physiologic adaptations, such as insulin growth signaling and the onset of insulin resistance [45], taking place at these early stages. Interestingly, studies in obese children have also reported increased LV radial strain [46] and torsion [40, 46] compared to lean counterparts. Additional studies are needed to determine if these findings are linked and, if so, the exact mechanisms underlying those responses.

### Relationship of mechanics to obesity sequelae

Fasting glucose levels and peripheral glucose clearance (AUC) were significantly altered (higher) in the obese group, indicative of glucose intolerance. These changes occurred very acutely after introduction of the diet, as early as 3 weeks, and these differences persisted and expanded during the course of the study. Prior studies have similarly reported acute changes in glucose tolerance [45]. The confounding role of insulin through interactions with β_2_ adrenergic receptors in the heart has already been noted [43]. Additionally, a recent study reported a therapeutic benefit of a glucagon-like peptide-1 analog, which regulates glucose metabolism, in reversing cardiac dysfunction in mice [36]. Here, we observed an overall weak but statistically significant association between glucose clearance and peak radial strain (a similarly weak correlation of longitudinal strain with glucose clearance was not significant after adjusting for group membership). These results are supportive of continued investigations into the role of altered insulin and/or glucose signaling in modulating cardiac function in obesity and the potential for therapies targeting these pathways.

Using tail cuff measurements, no significant differences in systolic blood pressure were observed, so we could not reliably evaluate the association of blood pressure and cardiac mechanics in these mice. Previous studies using more sensitive telemetry measures of blood pressure observed differences in male mice at 16 weeks of high-fat feeding [47], so it is likely that there were differences, which the tail cuff was not sensitive enough to detect [48]. Ideally, future studies could employ concurrent telemetry and MRI measurements; however, such devices would first need to be made MR compatible.

Myocardial mass was significantly elevated in the obese group beginning at 16 weeks on the diet, which is temporally consistent with presence of elevated blood pressure via telemetry [47]. This ventricular remodeling had a significant negative association with peak radial strain, which makes sense given that a thickened myocardium has to deform less radially to eject blood. However, no similar associations were present with respect to longitudinal (after adjusting for group membership) or circumferential strains, so the observed mechanical dysfunction cannot be entirely explained by this ventricular remodeling.

**Fig. 10:**
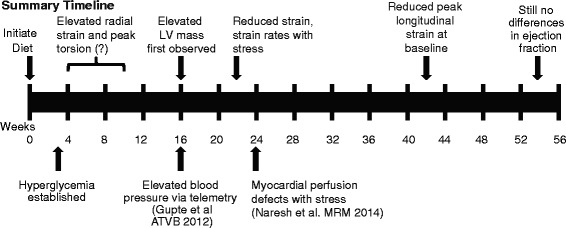
Summary timeline of study findings with respect to changes in cardiac mechanics and the development of obesity sequelae

Figure 10 presents a summary timeline of study findings with respect to impaired cardiac mechanics and their relationship to obesity sequelae.

### Inter-test and inter-observer reproducibility

The inter-test and inter-observer variability characteristics for DENSE-derived measures of strain, torsion, and synchrony in mice have been previously reported [49]. The DENSE-derived measures reported in this study were found to have acceptable reproducibility characteristics.

### Limitations

This study was conducted in mice, so the relevance of our findings with respect to changes in cardiac mechanics in obese humans, and particularly the relevant timing of the development of dysfunction, needs to be established. However, CMR can be acquired in both humans and mice, so there is a rare ability with this experimental protocol to make comparable measurements in human subjects and directly compare findings. Furthermore, elucidating the time course of dysfunction in a mouse model is important for future studies seeking to evaluate the effectiveness of clinically relevant treatment strategies for reversing this dysfunction in mice at meaningful time points.

Some of the mice used for these experiments were purchased through Jackson Laboratory’s Diet-Induced Obesity preconditioning service, which created some discrepancies in age and low-fat control diets. Specifically, mouse ‘cohorts’ 3–5 were purchased through the preconditioning service and were thus started on the diet at 6 weeks of age and the control diet was not sucrose-matched to the high-fat diet. Conversely, ‘cohorts’ 1–2 were started on their respective diets at 3 weeks of age and the control diet was sucrose-matched to the high-fat diet. Despite this variation, little, if any, differences were observed in the results between experiment 1 and baseline data acquired in experiment 2 (prior to the infusion of dobutamine) for weeks 4–16. Furthermore, the primary study findings were all based on data collected at or after 22 weeks on diet, at which point the ages and low-fat diets were consistent between experiments. Therefore, these differences had minimal effect on the study conclusions.

Finally, blood pressure data were collected using a tail cuff measurement, which has known limitations for detecting subtle differences in systolic pressure. Therefore, no quantitative comparison of pressure data to cardiac mechanics is presented here, even though increased afterload would be expected to affect cardiac function. Future studies should determine if pharmacologic treatment of hypertension in this model has beneficial effects with regard to altered cardiac mechanics as well.

## Conclusions

Diet-induced obesity in mice through one year of high-fat feeding results in increased left ventricular mass and altered left ventricular mechanics, but no significant change in left ventricular ejection fraction. These functional deficiencies in obesity were accentuated with pharmacologic stress, which revealed reduced peak strains, strain rates, and torsion of the left ventricle. Furthermore, these changes in mechanics were revealed at earlier stages of disease progression under stress conditions than at baseline. Along with quantification of ventricular mass, changes in mechanics may carry prognostic significance for adverse cardiovascular outcomes related to obesity—further study is needed to explore these links. Under both baseline and stress conditions, reduced peak longitudinal strain was a strong discriminator between obese and controls subjects. Therefore clinical evaluations of left ventricular function in obesity should quantify cardiac mechanics (strain, strain rate, torsion), particularly peak longitudinal strain, and may also benefit from stress imaging to better identify early signs of dysfunction.

## Additional file

Additional file 1:
**Presents Table S1-S9 (as referenced in text) containing summary statistics for all CMR mechanics measures and cardiac period.** (PDF 546 kb)

## Abbreviations

CMR: Cardiovascular magnetic resonance
DENSE: Displacement encoding with stimulated echoes
LV: Left ventricle/ventricular
AUC: Area under the curve
