# Supplementary material for: Left ventricular mechanical dysfunction in diet-induced obese mice is exacerbated during inotropic stress: a cine DENSE cardiovascular magnetic resonance study
Source: J Cardiovasc Magn Reson. 2015 Aug 27;17(1):75. doi: 10.1186/s12968-015-0180-7 (PMC4551701; doi:10.1186/s12968-015-0180-7)
Supplement: Additional file 1: — Presents Table S1-S9 (as referenced in text) containing summary statistics for all CMR mechanics measures and cardiac period. (PDF 546 kb) [file 12968_2015_180_MOESM1_ESM.pdf]

Table S1. Average Peak Left Ventricular Strain [%] at Baseline

Weeks on diet	Peak E_{rr}, Control		Peak E_{rr}, Obese		Peak E_{cc}, Control		Peak E_{cc}, Obese		Peak E_{ll}, Control		Peak E_{ll}, Obese	
	Mean	St. Dev	Mean	St. Dev	Mean	St. Dev	Mean	St. Dev	Mean	St. Dev	Mean	St. Dev
**4**	37.6	6.5	34.8	5.5	14.3	1.2	14.2	1.0	12.7	1.0	12.2	1.0
**10**	36.7	6.1	41.2	4.9	14.0	1.4	14.7	1.0	11.2	1.4	12.0	1.2
**16**	43.0	6.5	41.8	8.4	14.5	1.2	15.1	1.1	13.5	0.9	12.5	0.7
**22**	41.1	4.4	39.3	5.7	14.8	0.8	14.7	1.3	12.7	1.3	11.8	1.3
**28**	40.9	5.0	35.3	3.7	15.2	1.0	14.1	1.1	12.2	1.6	11.5	1.5
**34**	37.2	7.0	35.2	4.8	14.9	1.1	14.4	0.9	11.7	1.3	11.4	0.9
**42**	33.0	2.6	30.4	3.0	14.4	0.8	14.6	1.2	12.1	0.8	10.6	1.3
**54**	40.5	6.1	34.0	4.4	14.5	1.6	13.4	0.7	12.8	1.6	10.0	1.4

E_{rr} – radial strain, E_{cc} – circumferential strain, E_{ll} – longitudinal strain

Table S2. Cardiac period [ms] during scanning

Weeks on diet	Control, Baseline		Obese, Baseline		Control, Stress		Obese, Stress	
	Mean	St. Dev	Mean	St. Dev	Mean	St. Dev	Mean	St. Dev
**4**	134.2	9.5	131.1	11.71	100.6	3.0	101.9	5.5
**10**	129.9	7.8	128.5	15.3	104.2	6.2	107.1	4.3
**16**	146.5	9.1	147.6	10.2	106.3	4.6	107.2	4.6
**22**	136.6	12.0	144.6	19.5	106.2	5.4	106.1	4.9
**28**	144.2	14.3	144.0	16.9	103.7	5.2	107.1	3.6
**34**	133.2	15.8	140.1	18.9	N/A	N/A	N/A	N/A
**42**	130.4	11.5	129.0	15.2	N/A	N/A	N/A	N/A
**54/55**	131.7	12.1	123.4	18.5	104.1	6.0	110.1	5.3

Table S3. Average Peak Left Ventricular Systolic Strain Rate [%/ms] at Baseline

Weeks on diet	$\partial E_{rr}/\partial t$, Control		$\partial E_{rr}/\partial t$, Obese		$\partial E_{cc}/\partial t$, Control		$\partial E_{cc}/\partial t$, Obese		$\partial E_{ll}/\partial t$, Control		$\partial E_{ll}/\partial t$, Obese	
	Mean	St. Dev	Mean	St. Dev	Mean	St. Dev	Mean	St. Dev	Mean	St. Dev	Mean	St. Dev
**4**	1.12	0.25	1.07	0.25	0.51	0.05	0.54	0.06	0.49	0.12	0.51	0.08
**10**	1.19	0.19	1.35	0.22	0.54	0.05	0.58	0.05	0.46	0.07	0.48	0.04
**16**	1.14	0.17	1.09	0.19	0.52	0.06	0.51	0.04	0.51	0.08	0.45	0.08
**22**	1.16	0.19	1.10	0.15	0.53	0.05	0.50	0.06	0.45	0.06	0.41	0.04
**28**	1.05	0.11	1.00	0.16	0.52	0.07	0.50	0.04	0.46	0.07	0.50	0.14
**34**	1.13	0.27	1.00	0.13	0.55	0.05	0.53	0.05	0.42	0.05	0.50	0.26
**42**	1.04	0.19	0.99	0.11	0.56	0.06	0.55	0.02	0.50	0.08	0.44	0.07
**54**	1.24	0.28	1.15	0.28	0.52	0.05	0.50	0.03	0.49	0.08	0.43	0.10

$\partial E_{rr}/\partial t$ – radial strain rate, $\partial E_{cc}/\partial t$ – circumferential strain rate, $\partial E_{ll}/\partial t$ – longitudinal strain rate

Table S4. Average Peak Left Ventricular Diastolic Strain Rate [%/ms] at Baseline

Weeks on diet	$\partial E_{rr}/\partial t$, Control		$\partial E_{rr}/\partial t$, Obese		$\partial E_{cc}/\partial t$, Control		$\partial E_{cc}/\partial t$, Obese		$\partial E_{ll}/\partial t$, Control		$\partial E_{ll}/\partial t$, Obese	
	Mean	St. Dev	Mean	St. Dev	Mean	St. Dev	Mean	St. Dev	Mean	St. Dev	Mean	St. Dev
4	-1.15	0.26	-1.00	0.23	-0.39	0.06	-0.39	0.05	-0.34	0.09	-0.34	0.09
10	-1.09	0.22	-1.25	0.25	-0.39	0.04	-0.43	0.06	-0.26	0.06	-0.32	0.08
16	-1.27	0.26	-1.21	0.30	-0.36	0.04	-0.41	0.06	-0.35	0.06	-0.28	0.06
22	-1.23	0.23	-1.14	0.27	-0.41	0.05	-0.43	0.09	-0.35	0.05	-0.29	0.08
28	-1.22	0.14	-1.01	0.23	-0.43	0.12	-0.42	0.09	-0.35	0.07	-0.33	0.12
34	-1.18	0.34	-1.01	0.19	-0.44	0.11	-0.44	0.07	-0.34	0.11	-0.34	0.08
42	-1.09	0.17	-1.02	0.14	-0.43	0.06	-0.45	0.07	-0.35	0.12	-0.29	0.11
54	-1.24	0.36	-1.16	0.32	-0.44	0.08	-0.47	0.07	-0.40	0.09	-0.45	0.23

Table S5. Peak Left Ventricular Torsion [°/cm] at Baseline

Weeks on diet	Control		Obese	
	Mean	St. Dev	Mean	St. Dev
4	4.0	0.4	4.0	0.6
10	3.6	0.6	3.9	0.4
16	4.1	0.5	3.7	0.6
22	3.9	0.7	3.6	0.4
28	4.1	0.4	3.5	0.7
34	4.1	0.4	3.9	0.6
42	4.0	0.5	3.6	0.4
54	4.0	0.7	3.8	0.6

Table S6. Average Peak Left Ventricular Strain [%] at Stress

Weeks on diet	Peak E_{rr}, Control		Peak E_{rr}, Obese		Peak E_{cc}, Control		Peak E_{cc}, Obese		Peak E_{ll}, Control		Peak E_{ll}, Obese	
	Mean	St. Dev	Mean	St. Dev	Mean	St. Dev	Mean	St. Dev	Mean	St. Dev	Mean	St. Dev
4	34.6	5.4	41.8	5.4	15.7	2.1	15.9	1.3	12.1	0.8	12.4	1.3
10	42.5	4.7	40.2	6.5	16.3	0.7	17.2	1.4	11.8	0.6	12.4	1.3
16	46.4	5.3	43.8	4.8	15.4	1.5	15.8	1.3	11.8	1.3	12.2	1.9
22	47.8	5.0	43.1	5.1	15.9	1.0	16.0	1.5	12.5	1.1	10.8	1.7
28	47.1	6.6	43.5	9.6	16.3	1.4	16.4	1.3	11.9	1.3	11.1	1.6
55	41.2	4.0	34.6	4.8	16.4	0.9	14.8	1.7	11.5	1.8	9.6	1.8

Table S7. Average Peak Left Ventricular Systolic Strain Rate [%/ms] at Stress

Weeks on diet	$\partial E_{rr}/\partial t$, Control		$\partial E_{rr}/\partial t$, Obese		$\partial E_{cc}/\partial t$, Control		$\partial E_{cc}/\partial t$, Obese		$\partial E_{ll}/\partial t$, Control		$\partial E_{ll}/\partial t$, Obese	
	Mean	St. Dev	Mean	St. Dev	Mean	St. Dev	Mean	St. Dev	Mean	St. Dev	Mean	St. Dev
**4**	1.40	0.28	1.56	0.30	0.74	0.11	0.73	0.11	0.61	0.10	0.60	0.11
**10**	1.63	0.30	1.46	0.28	0.77	0.06	0.76	0.10	0.60	0.12	0.61	0.11
**16**	1.83	0.23	1.70	0.20	0.75	0.06	0.70	0.03	0.62	0.09	0.63	0.11
**22**	1.98	0.20	1.61	0.19	0.77	0.04	0.68	0.02	0.63	0.05	0.52	0.07
**28**	1.93	0.22	1.68	0.31	0.78	0.05	0.68	0.04	0.60	0.07	0.54	0.11
**55**	1.60	0.26	1.20	0.16	0.78	0.06	0.60	0.10	0.62	0.11	0.48	0.14

Table S8. Average Peak Left Ventricular Diastolic Strain Rate [%/ms] at Stress

Weeks on diet	$\partial E_{rr}/\partial t$, Control		$\partial E_{rr}/\partial t$, Obese		$\partial E_{cc}/\partial t$, Control		$\partial E_{cc}/\partial t$, Obese		$\partial E_{ll}/\partial t$, Control		$\partial E_{ll}/\partial t$, Obese	
	Mean	St. Dev	Mean	St. Dev	Mean	St. Dev	Mean	St. Dev	Mean	St. Dev	Mean	St. Dev
**4**	-1.15	0.35	-1.69	0.33	-0.61	0.12	-0.58	0.06	-0.49	0.15	-0.48	0.08
**10**	-1.54	0.31	-1.46	0.26	-0.57	0.08	-0.60	0.10	-0.49	0.15	-0.40	0.10
**16**	-1.87	0.26	-1.63	0.24	-0.52	0.06	-0.55	0.05	-0.54	0.10	-0.38	0.15
**22**	-1.96	0.24	-1.77	0.31	-0.57	0.05	-0.63	0.14	-0.49	0.13	-0.43	0.12
**28**	-1.87	0.29	-1.77	0.40	-0.60	0.07	-0.65	0.11	-0.48	0.13	-0.42	0.16
**55**	-1.48	0.30	-1.20	0.24	-0.57	0.08	-0.54	0.09	-0.51	0.17	-0.36	0.10

Table S9. Peak Left Ventricular Torsion [°/cm] at Stress

Weeks on diet	Control		Obese	
	Mean	St. Dev	Mean	St. Dev
**4**	5.0	1.3	5.6	0.7
**10**	5.1	0.6	6.1	1.3
**16**	5.0	1.0	5.1	0.4
**22**	5.8	0.7	5.2	0.4
**28**	5.8	0.7	5.2	0.7
**55**	5.8	0.8	4.1	0.6
